# Application of virtual simulation in clinical skills and operation courses

**DOI:** 10.3389/fmed.2023.1184392

**Published:** 2023-05-25

**Authors:** Li Wang, Feng Zhang, Hongxiang Xie

**Affiliations:** ^1^School of Clinical Medicine, Hangzhou Medical College, Hangzhou, Zhejiang, China; ^2^Laboratory Medicine Center, Department of Clinical Laboratory, Zhejiang Provincial People's Hospital, Affiliated People's Hospital, Hangzhou Medical College, Hangzhou, Zhejiang, China

**Keywords:** virtual simulation, clinical skills and operation, clinical medicine course, online teaching, COVID-19

## Abstract

**Aim:**

This study investigated the effectiveness and prospect of applying virtual simulation operation (VSO) as a novel teaching tool in clinical skill and operation courses.

**Methods:**

A comparative test and survey study on the teaching effect of VSO was conducted with the clinical skill and operation course as the test course. The test group students received offline courses combined with online VSO practice. In contrast, the control group students received offline courses combined with instructional video review. The two groups were assessed using the Chinese medical school clinical medicine professional level test and a questionnaire survey.

**Results:**

The test group students scored significantly higher than the control group in the skills test (score difference: 3.43, 95% CI: 2.05–4.80) (*p* < 0.001). Additionally, a significant increase in the percentage of high-and intermediate-score ranges and a decrease in the percentage of low-score ranges was observed (*p* < 0.001). According to the questionnaire survey, 80.56% of the students were willing to continue using virtual simulation in their subsequent clinical skill and operation learning. Further, 85.19% of the students believed that the VSO is superior because it is unrestricted by time and space and can be performed anywhere and anytime compared to traditional operation training.

**Conclusion:**

VSO teaching can improve skills and examination performance. An entirely online operation that does not need special equipment can break through the spatiotemporal limitations of traditional skills courses. VSO teaching also suits the ongoing COVID-19 pandemic situation. Virtual simulation, a new teaching tool, has good application prospects.

## 1. Introduction

We have focused on developing a virtual simulation program for clinical skills since 2013 and established an online platform called “Virtual Hospital.” The virtual simulation operation (VSO) played a key role during COVID-19, owing to the requirement of conducting online classes. Accordingly, the virtual simulation program entered a new stage of development.

Using computer technology, virtual simulation generates virtual scenes, instruments, characters, and experimental animals based on real teaching content, allowing teachers and students to engage in a virtual-reality environment with computers, cell phones, and other terminal devices. Virtual simulation operation (VSO) can effectively avoid the risk entailed in real operations and is characterized by simulation, interaction, and openness (1). Clinical medicine has its features compared to other disciplines because its object of study is the human body. Additionally, most operations involving real people are risky. Therefore, the development of VSO teaching programs is particularly suitable for clinical medicine (2).

The rapid advancement of information technology has significantly changed the underlying technology of virtual simulation. There is also an increasing demand for teaching clinical skills in medical schools. Particularly, COVID-19 led to a surge in demand for online teaching. Virtual simulation, based on networking, is particularly suitable for online teaching (3). Our college started to explore the development of a VSO teaching program as early as 2013. We have always been dedicated to the construction of high-quality virtual simulation programs, from the initial FLASH to today’s cross-terminal platform based on HTML5 and UNITY. We also extensively collected user feedback during the development process, which laid the foundation for future improvement and optimization.

**Figure 1:**
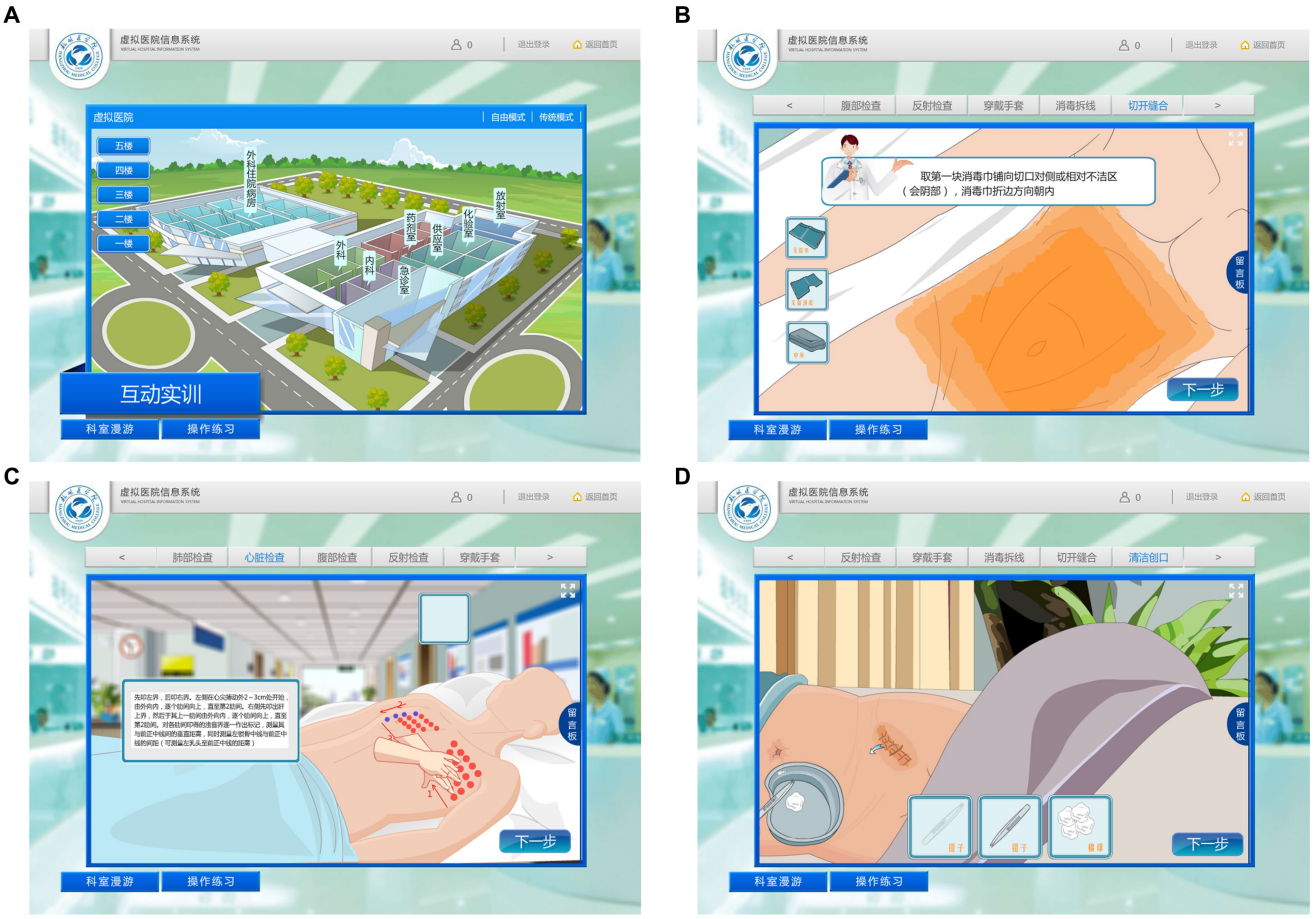
“Virtual Hospital” information system. **(A)** Appearance scene of the virtual hospital. **(B)** Surgical patient disinfection and surgical drape operations. **(C)** Heart percussion operation. **(D)** Surgical dressing-change operation.

Mobile Internet has penetrated all aspects of life. However, conducting VSO teaching programs before 2014 (3G era) was impossible due to the limitations of cell phone performance, network speed, and cost (4). Therefore, the personal computer (PC) was the mainstream teaching terminal device at that time. Additionally, FLASH was the most used development software owing to its superiorities such as low development cost, low difficulty, and cross-terminal operation (5). The first attempt to develop a VSO program using FLASH in our college was the “Virtual Hospital” information system (Figure 1). The feedback from students was very positive after the system went live. Most students felt that the VSO teaching program was novel and could stimulate their interest in learning. The research team designed a questionnaire on their own based on previous survey practices (6) and after discussion with our pedagogical experts, in conjunction with this study. The questionnaire was statistically calibrated and had reasonable reliability and validity. The questionnaire survey revealed that 93% of the students found the virtual simulation teaching to be novel. The students’ objective structured clinical skills examination scores also improved significantly after the application of the “Virtual Hospital” to assist teaching.

However, the backward screen performance, unreasonable use of equipment resources, and software vulnerabilities led to the gradual withdrawal of FLASH from the historical stage. In 2020, Adobe, the parent company of FLASH, announced that it would stop supporting Flash Player, starting from December 31, 2020. Its successor, HTML5, with its exceptional superiority, could completely replace FLASH in all aspects and become the mainstream web technology language in the new era (7).

The “Virtual Hospital V2.0” system has been basically developed. The system, developed based on HTML5 and UNITY, has completely surpassed its old version in terms of screen performance and user experience, and it has achieved multi-terminal coverage. In the era of mobile Internet, smartphones have become the most frequently used terminal by students. Therefore, the new version of the system has been optimized for compatibility with cell phones.

**Figure 2:**
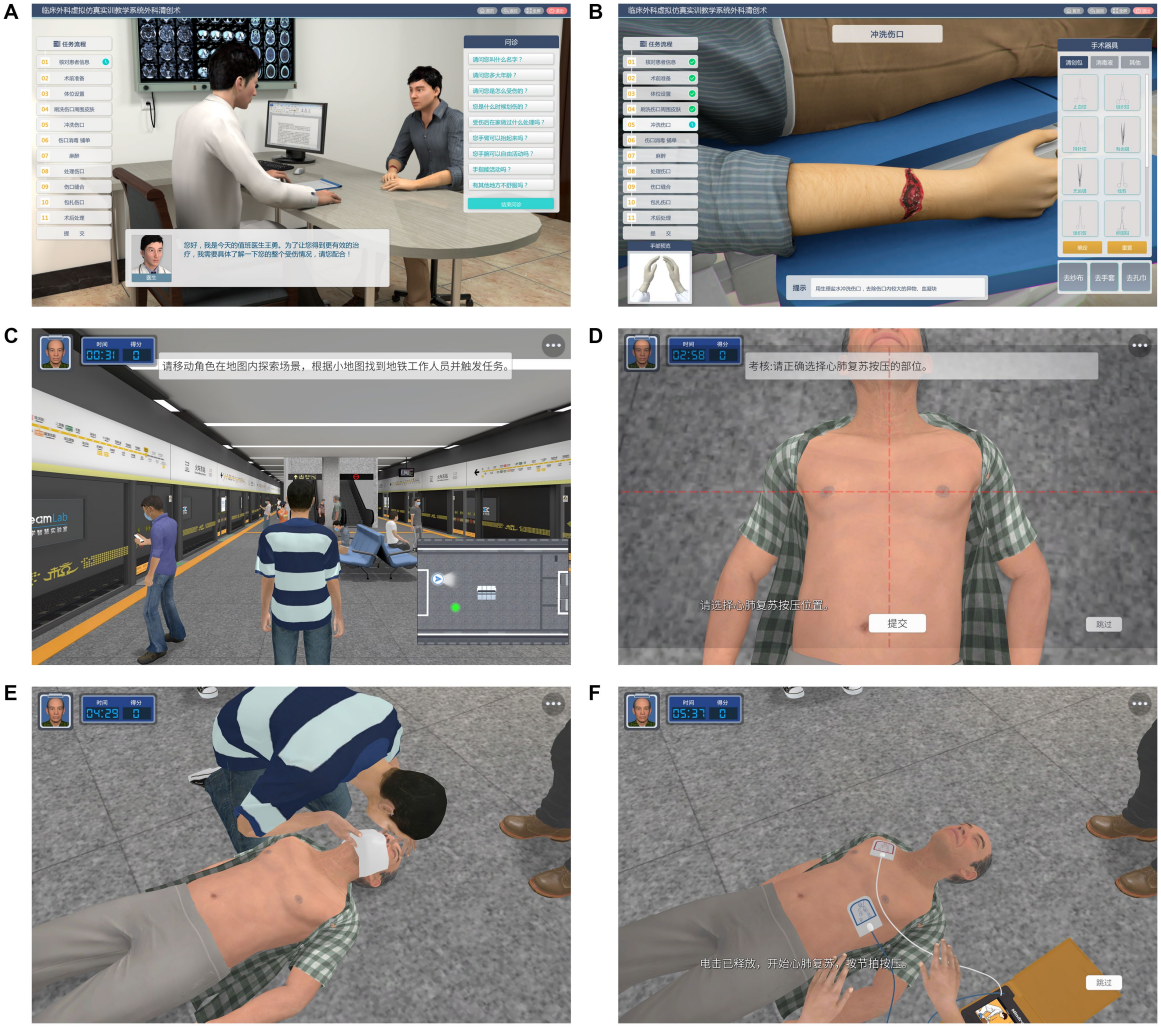
“Virtual Hospital V2.0” information system. **(A)** Simulated consultation. **(B)** Debridement operation. **(C)** Resuscitation scene of a cardiac arrest patient in a subway station. **(D)** External chest compression (select compression site). **(E)** Airway opening and artificial respiration. **(F)** AED operation.

Virtual simulation technology is very mature in commercial games, in which students have been exposed to very realistic and high-end game graphics. However, there is presently a gap between the graphic expressiveness of the virtual simulation software for teaching and commercial games due to the former’s non-profit nature (8). Therefore, some students have a comparison mentality when using virtual simulation teaching software and are dissatisfied with its poor quality and rough graphics. However, technological progress makes virtual graphics increasingly realistic. Accordingly, the virtual simulation program screen for teaching is also becoming increasingly exquisite. Some foreign teaching teams have directly been applying the commercial game engine Unreal Engine 5 to develop teaching programs (8, 9). While developing the “Virtual Hospital V2.0” software, more attention has been paid to the quality of graphics and ambient light rendering. Additionally, the three-dimensional (3D) human modeling is more proximate to the real human data, and the action screen is more exquisite and smoother (Figure 2).

Unlike augmented reality (AR) and virtual reality (VR) simulation, the VSO is fully online and does not require additional special equipment. Therefore, teachers and students can use the system anytime and anywhere, only with a terminal device that can be connected to the Internet.

This study discussed the role of VSO in teaching clinical skills based on a teaching reform experiment conducted in a recent teaching cycle. Further, the future trends of virtual simulation teaching and learning were also explored.

## 2. Methodology

Teaching effectiveness has been repeatedly observed and tested in the ongoing development of the virtual simulation skill practice program. A comparative study was conducted in a recent teaching cycle in which four classes were randomly selected as the test group (*n* = 108) and the remaining four classes as the control group (*n* = 116), using a random number table method from a total of eight classes (*n* = 224) of students in the same year of clinical medicine. The test course was a clinical skills and operation course (course ID: 39 AC032) involving clinical skills operations related to internal medicine, surgery, obstetrics and gynecology, pediatrics, and emergency medicine (Appendix 1). This was consistent with the operational assessment items of the Chinese medical school clinical medicine professional (undergraduate) level test. The test group students received offline courses combined with VSOs. They used both the medical model for practice and the online VSOs for review. The research group’s studies were all based on the premise that the normal teaching content and order would not be affected, the school’s talent training program and syllabus would not be violated, and reasonable compliance would be achieved before considering the use of VSO teaching tools for teaching. Moreover, we conducted a survey of the students in the experimental group in the course of conducting the study and analyzed the feedback in a timely manner. Participants were notified that their participation was voluntary and that they could choose to end their participation at anytime during the training.

In contrast, the control group students received an all-offline course. They practiced with the medical model and reviewed using instructional videos. The baseline analysis compared the age, gender ratio, China national college entrance exam (CNCEE) scores, and prior clinical course scores of the students in both groups; no statistical differences were observed (*p* > 0.05). All students had the same prior course curriculum and the same faculty. After one complete teaching cycle, the performance of both groups was compared using the Chinese medical school clinical medicine professional (undergraduate) level test as an evaluation index. The assessment items of this test include clinical skills and operations related to internal medicine, surgery, obstetrics and gynecology, pediatrics, and emergency medicine, which are consistent with the items taught in the test course. Further, a questionnaire with a reliability Cronbach’s 𝛼 coefficient of 0.905 and a validity Kaiser-Meyer-Olkin Measure of Sampling Adequacy (KMO) value of 0.879 was distributed to the test group students at the end of the semester. In total, 108 questionnaires were collected, with an efficiency rate of 100%.

Quantitative data were tested for normality using the Shapiro–Wilk method, and normal data were expressed as mean ± standard deviation and compared by *t*-test. Skewed data were expressed as median (p25, p75) and compared using the Wilcoxon Mann–Whitney test. Categorical data were compared using the Chi-squared test or Fisher’s exact test, and the score differences were analyzed using the Hodges-Lehmann method. Additionally, *p* < 0.05 suggested statistical significance. All statistical analyses were performed using SPSS25.

## 3. Results

Baseline analyses were performed on both groups to ensure comparability, including age, sex, CNCEE scores, and average scores in prior clinical courses. No statistical differences were found between the two groups in terms of these variables (*p* > 0.05; Table 1).

**Table 1:** Baseline analysis results.

	Test group	Control group	Statistical value	*p*-value
Age (in years)	22 (22,23)	22 (22,23)	*Z* = −1.437	0.151
Sex	Male 49 (45.4) Female 59 (54.6)	Male 56 (48.3) Female 60 (51.7)	*χ*^2^ = 0.19	0.689
CNCEE scores	604 (597, 610)	603 (596, 609)	*Z* = −0.649	0.517
Average score in prior clinical courses	77 (68, 83)	77 (71, 87)	*Z* = −1.103	0.27

The Shapiro–Wilk method-based normality test indicated skewed score distribution (*p* < 0.05) in both groups. The median scores of the test and control groups were 84.08 (80.71, 87.59) and 81.27 (76.35, 83.97), respectively. Statistical differences were observed in the distribution of scores between the two groups (*Z* = −4.852, *p* < 0.05). The test group students scored significantly higher than the control group students in the skills examination, with a difference of 3.43 (2.05–4.80) points (Table 2).

**Table 2:** Comparative analysis of the Chinese medical school clinical medicine professional (undergraduate) level test.

Group	M (*P*_25_, *P*_75_)	Median of the difference (95% CI)	Wilcoxon Mann–Whitney test	
			*Z*	*P*
Test group	84.08 (80.71, 87.59)	3.43 (2.05–4.80)	−4.852	<0.001
Control group	81.27 (76.35, 83.97)			

Constituent ratio analysis was conducted on the scores of both groups, where scores <80 were defined as a low-score range, 80–90 as an intermediate-score range, and > 90 as a high-score range. Fisher’s exact test analysis indicated a 6.5 and 0.0% of high-score range for the test and control groups, respectively. Thus, significant differences (*p* < 0.05) were observed in the distribution of score ranges between the two groups. Using post-hoc tests (bonferroni), we found significant differences between the <80 score band and the >90 score band in both groups (Table 3).

**Table 3:** Comparative analysis of score constituent ratio.

Group	Total (*n*)	Low-score range (<80) [*n* (%)]	Intermediate-score range (80–90) [*n* (%)]	High-score range (>90) [*n* (%)]	Fisher’s exact test
					*P*
Test group	108	25 (23.1)*	76 (70.4)	7 (6.5)*	<0.001
Control group	116	47 (40.5)	69 (59.5)	0 (0.0)	

* Statistically significant difference compared with the control group (*p* < 0.05).

According to the questionnaire survey, 68.52% of the students had not been exposed to similar VSOs before. Additionally, 85.19% of the students believed that the VSO’s superiority lies in the fact that it is unrestricted by time and space and can be conducted anywhere and anytime compared to traditional operation training. Moreover, 62.96% of the students believed that VSOs can provide equipment that is difficult to access in reality. However, 71.3% of the students stated that the extent of virtual simulation is not high, and it differs from real clinical operations. Additionally, 58.33% of the students complained that VSOs cannot be conducted on real patients, which is not helpful for hands-on practice. Overall, 80.56% of the students wanted to continue using virtual simulation in their subsequent clinical skills learning (Table 4).

**Table 4:** Main results of the questionnaire survey (*N* = 108).

Questionnaire item	Agree (*n* [%])
*Superiority*	
It is not limited by time and space and can be conducted anywhere	92 (85.19)
VSOs can provide equipment that is difficult to access in reality	68 (62.96)
Repeat training is possible with no consumable loss and is environmentally friendly	67 (62.04)
*Weakness*	
The extent of virtual simulation is not high with some differences from real clinical operations	77 (71.30)
VSOs cannot be conducted on real patients, which is not helpful for hands-on practice	63 (58.33)
The interactivity of the simulation platform is still lacking, and there is still room for improvement in the user experience	48 (44.44)

**Figure 3:**
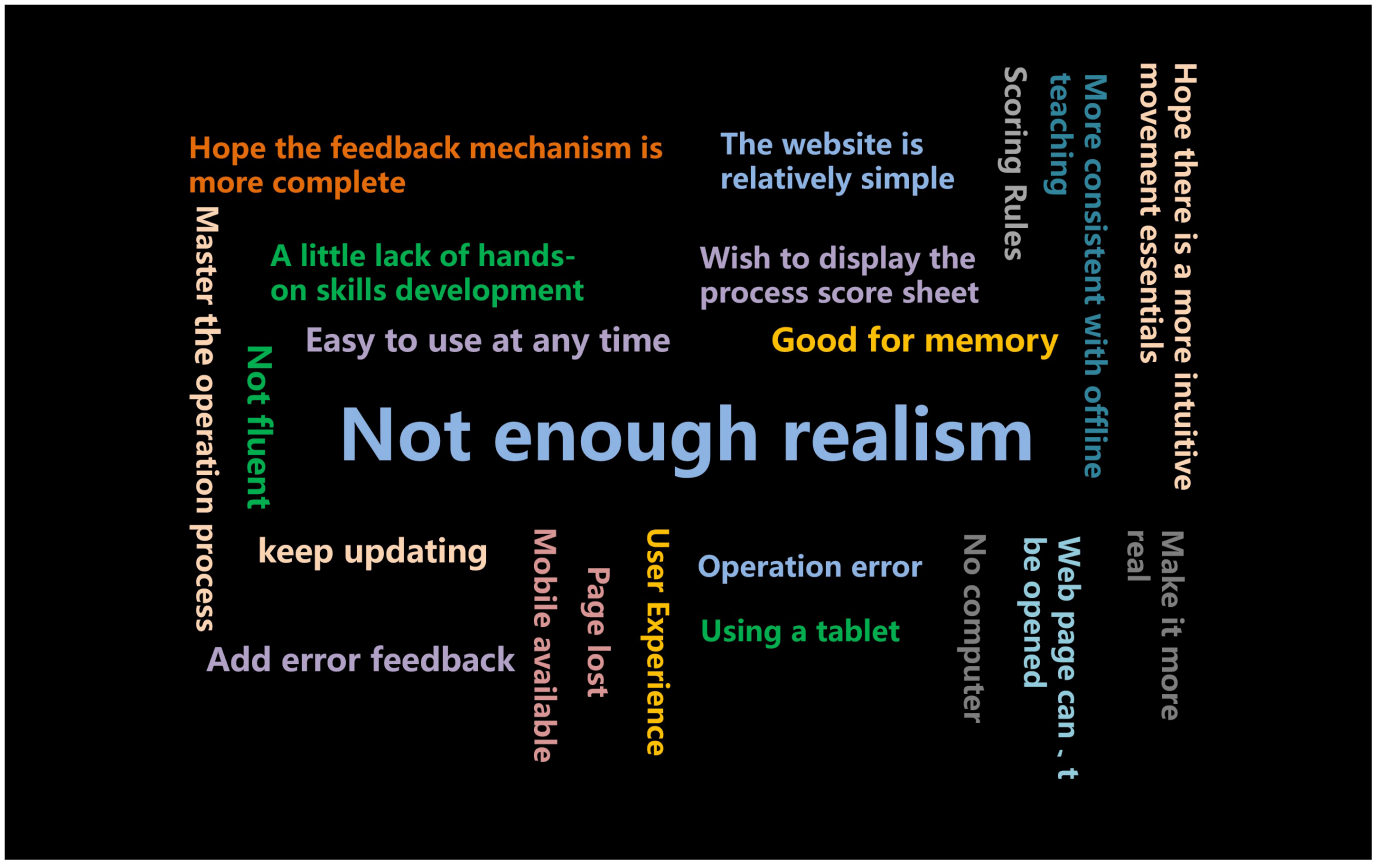
Hot word map of students’ opinions on VSOs in the questionnaire. The more middle the position the words and phrases, the higher the present frequency in the questionnaire.

The last item of the questionnaire was an open-ended question asking students about their experience with VSO and suggestions for improvement. Based on the responses collected from the survey, a hot word map was created, in which the more central the position of words and phrases, the more frequently they appeared in the responses (Figure 3).

## 4. Discussion

The COVID-19 pandemic ravaged the whole world in early 2020 and had a significant negative impact on teaching and learning in higher education. Online teaching has become the preferred contingency solution for schools to ensure the proper delivery of teaching assignments due to the hindrances to traditional face-to-face teaching (10). The virtual simulation of clinical skills based on the Internet is available to students and faculty with devices that have access to the Internet. Therefore, VSO teaching is an alternative to traditional offline clinical skills courses (11).

Compared to theoretical courses, teaching clinical skills operation courses online is more difficult because students cannot engage in actual operation training online. Virtual simulation with the Internet is apt for the current teaching needs. The introduction of various new information technologies, such as 3D display and perspective view of the internal structure of the human body, can present richer teaching materials and teaching effects than traditional offline courses; these also entail high-quality online teaching tools for clinical skills courses (12).

The present teaching experiment corroborated the idea that VSO contributes to high-quality engagement in clinical skill courses. The test group students scored significantly higher than the control group students in the skills examination, and it has increased the number of students in the high-scoring band (>90) and reduced the number of students in the low-scoring band (<80). This indicates that in this trial, VSOs can improve students’ skill assessment scores relatively evenly, rather than working for only some students. This indicates that students who used VSO increased their skill assessment scores compared to those who did not, with other teaching tools and content remaining the same, so we believe that VSO can improve students’ clinical skill operations and is a new promising teaching tool. A good teaching tool, in addition to being effective, should also be enjoyed by students. According to the questionnaire survey, most students were highly receptive to virtual simulation. Furthermore, lots of the students believed that the VSO’s most prominent superiority is that it is not limited by time, space, and equipment, which is consistent with our original intention of developing the VSO program.

The ultimate purpose of the VSO teaching program is to serve the curriculum, which is the core element of talent training. The promotion of teaching reforms with information technology is precisely the entry point of VSO teaching program development in the construction of high-quality clinical courses. VSO, together with online open courses and online and offline hybrid courses, should be used as a tool to promote the construction of first-class courses and create a multi-dimensional medical curriculum system. Particularly, clinical skills courses are more suitable to be built as online and offline hybrid courses. During the COVID-19 pandemic, building clinical skills courses into an online-offline hybrid form, wherein medical models are used in offline teaching and virtual simulation is used in online teaching, can solve the teaching problems of clinical skills courses more perfectly; thus, this teaching reform direction should be extensively promoted (13).

The construction of large-scale open online massive open online courses (MOOCs) is a very successful model in the teaching reform of theory courses (14). Drawing on the successful experience of MOOC, an important construction direction of VSO teaching is shared construction. The funds and efforts required for VSO teaching programs are incomparable to those of theoretical courses. Therefore, it is difficult to establish a complete and program-complete virtual simulation teaching system in one institution alone. The construction of the VSO teaching platform and sharing of teaching resources and network resources with partner institutions worldwide can ensure the maximum utilization of resources, which is a necessary way for virtual simulation course construction (15).

COVID-19 has facilitated the “internetization” of traditional courses. VSO program construction has also ushered in a good time to accelerate the construction and promote the application in the general environment of comprehensive construction of a first-class curriculum. The VSO teaching program, which has an Internet attribute, can replace the traditional clinical skills course under the new mode of online teaching to fully tap the sensory perception of students to participate in the VSOs. Therefore, it is a teaching tool that is in line with the trend of the times and has a broad development prospect (16). The construction of the current VSO program has taken shape. Integrating VSOs more deeply into the curriculum and creating a first-class VSO course will be a new trend in the reform of clinical medicine teaching (17).

This study verified the effectiveness of VSO through a comparison study and also gained the approval of students, but there are still shortcomings in this study. The current VSO program still has limitations. According to the questionnaire survey and hot word map, most students believed that the extent of simulation in the current virtual simulation program is not high enough and vastly differs from reality. Additionally, VSO is a purely screen-based operation, which is not conducive to the development of hands-on skills. Some students even complained that “the website is still a bit rudimentary.” Students have high expectations regarding the quality of virtual simulation programs. Therefore, the developers of VSO programs should invest more efforts and funds to create more exquisite and higher-quality VSO programs.

In addition, the result showed that the test group students scored significantly higher than the control group, raising questions about the ethics of withholding the VSO intervention from the control group. In fact, the research team had applied to the Medical Ethics Committee for ethical review, but our application was not accepted on the grounds that the study was not a biomedical study and the Declaration of Helsinki did not apply and that the content and quality of the lectures. However, we fully informed all students before administering the VSO teaching method. We explained that there were also risks in the experimental group because it was not certain before the trial that the VSO teaching method would actually be effective. If there were unwilling students after informing them, they were placed in traditional teaching classes, but their performance did not enter into the comparison of experimental data. We conducted random sampling on the basis of informed consent of all. In practice, all students participated voluntarily in this teaching study. In addition, both of the experimental and control groups met the requirements of the university’s talent training program and syllabus, and complied with our laws and relevant industry regulations at the policy level.

The study team suggests continuing to improve the quality of VSOs and promoting online teaching models for clinical skills practice courses to be ready for special situations where offline teaching cannot be conducted.

## Data availability statement

The raw data supporting the conclusions of this article will be made available by the authors, without undue reservation.

## Ethics staement

Ethical review and approval was not required for the study on human participants in accordance with the local legislation and institutional requirements. Written informed consent for participation was not required for this study in accordance with the national legislation and the institutional requirements.

## Author contributions

LW: funding acquisition and writing of the original draft. FZ: analyzed the data and revising the manuscript. HX: research implementation guidance and manuscript revision. All authors have accepted responsibility for the entire content of this manuscript and approved its submission.

## Funding

This research was funded by the Virtual Simulation Experimental Teaching Program for Universities in the Thirteen Five-Year Plan of Zhejiang Province [Zhe Jiao Ban Han (2019) No. 365, funding organizations: Education Department of Zhejiang Province, China] to LW. Science and Technology Project of Medicine and Health of Zhejiang (2021KY017 and 2022KY027), General Research Project of the Education Department of Zhejiang Province (Y202146133), Traditional Chinese Medicine Science and Technology Project of Zhejiang Province (2023ZL009), and Basic Scientific Research Funds of Department of Education of Zhejiang Province (KYYB202216) to HX.

## Conflict of interest

The authors declare that the research was conducted in the absence of any commercial or financial relationships that could be construed as a potential conflict of interest.

## Publisher’s note

All claims expressed in this article are solely those of the authors and do not necessarily represent those of their affiliated organizations, or those of the publisher, the editors and the reviewers. Any product that may be evaluated in this article, or claim that may be made by its manufacturer, is not guaranteed or endorsed by the publisher.

## Appendix 1

Catalog of clinical skills and operation items:

1. Physical examination

2. Thoracentesis

3. Celiocentesis

4. Bone marrow aspiration

5. Lumbar puncture

6. Pre-operative aseptic preparation by surgeon

7. Sterile preparation of the patient’s surgical area

8. Incision and suturing

9. Debridement

10. CPR and AED

11. Obstetrical and gynecological examination

12. Gastric tube insertion

13. Urinary catheterization

14. Intravenous injection

15. Putting on and taking off isolation gown
